# A turn-on AIE sensor for nanomolar detection of perrhenate in aqueous media

**DOI:** 10.1039/d6ra01192f

**Published:** 2026-03-26

**Authors:** Yan-ni Li, Yan-xin Du, Hao Liu, Yi-jie Zhu, Fan Deng, Qin-feng Xu

**Affiliations:** a School of Food Science and Engineering, National Research and Development Center for Goat Dairy Products Processing Technology, Shaanxi University of Science and Technology Xi'an Shaanxi 710021 China xuqinfeng@sust.edu.cn

## Abstract

The “light-switch” Ru(ii) polypyridine complexes have been developed as a novel fluorescent sensor for rapid and sensitive detection of ReO_4_^−^ anions in pure aqueous media. When counterions (Cl^−^) in aqueous solution are specifically exchanged by ReO_4_^−^ anions, the “light-switch” Ru(ii) polypyridine complexes exhibit a significant fluorescence emission enhancement, accompanied by the formation of nanoaggregates. This aggregation is driven by weak interactions with electrostatic attraction, anion⋯π, C–H⋯anion hydrogen bonding and π–π stacking interactions between ReO_4_^−^ anions and “light-switch” Ru(ii) polypyridine complexes, which is confirmed by single crystal structure analysis, density functional theory (DFT) and Energy decomposition analysis (EDA). The anion exchange-induced aggregation effect of the Ru(ii) polypyridine complexes demonstrate a potent “light-switch” strategy, capable of rapid response to ReO_4_^−^ within 1 s and a limit of quantification of 1.5 nM, below the WHO guideline value for its radioactive analogue ^99^TcO_4_^−^ (1.6 nM), demonstrating potential for monitoring pertechnetate contamination. This work opens a new way for future research based on “light-switch” Ru(ii) polypyridine complexes as turn-on fluorescent sensing probes for ReO_4_^−^/^99^TcO_4_^−^ anions detection in aqueous media.

## Abstract 2

The optimal complex, Ru3, achieves high sensitivity and selective in a “turn-on” luminescent response to ReO_4_^−^ in pure aqueous medium, with LOD of 3.4 nM.

## Graphical abstract

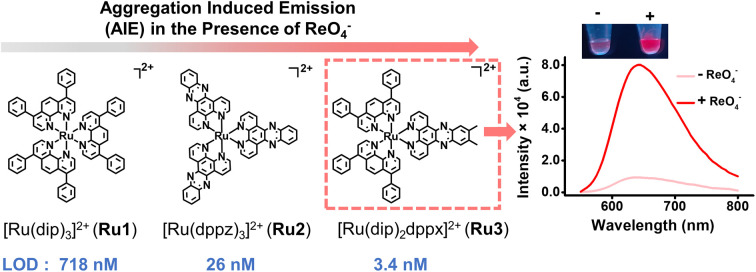

## 1 Introduction

Radioactive oxyanions, such as pertechnetate (TcO_4_^−^), a fission product of the nuclear industry, are highly mobile in the environment and can accumulate through the food chain, posing a persistent threat to human health and ecosystems.^1–3^ Therefore, the development of detection methods with high selectivity and sensitivity for TcO_4_^−^ is of urgent practical significance. Current mainstream quantitative techniques, such as accelerator mass spectrometry (AMS),^4^ surface-enhanced Raman spectroscopy (SERS),^5^ inductively coupled plasma mass spectrometry (ICP-MS),^6,7^ offer high sensitivity and accuracy but rely on bulky instrumentation and complex sample preparation, making them unsuitable for rapid on-site screening. Consequently, the development of convenient and sensitive fluorescence-based sensing technologies has become a vital complementary approach. Given the radioactivity of TcO_4_^−^, its chemical surrogate, perrhenate (ReO_4_^−^), which shares a similar ionic radius and tetrahedral geometry, is widely used in sensing mechanism studies.^8,9^

In recent years, significant progress has been made in fluorescence sensing technology for ReO_4_^−^/TcO_4_^−^ detection. This progress has primarily involved two sensor types: fluorescence “turn-off”^10–21^ and fluorescence “turn-on”.^22–29^ Compared to the former, “turn-on” sensors have garnered considerable attention due to their higher anti-interference capability and more intuitive signal output. Currently reported “turn-on” systems mainly fall into two categories: (i) organic molecules based on aggregation-induced emission (AIE),^24,29^ and (ii) sensing materials based on metal complexes, such as functionalized Ir(iii) porous aromatic frameworks,^27^ Ru complexes,^28^ and Pt complexes.^26^ Among these, certain Pt(II) complex-based probes have achieved high selectivity and sensitivity (LOD as low as 0.26 nM) for TcO_4_^−^ detection through mechanisms like anion exchange-induced Pt⋯Pt interactions and preconcentration using porous glass beads (*e.g.*, Vycor).^26^ However, the reliance on noble metal platinum and potentially toxic antimony-containing counterions ([Pt(tpy)Br]SbF_6_) in such systems poses limitations in terms of cost and environmental compatibility, restricting their prospects for large-scale application.

Alternatively, Ru(ii) polypyridine complexes have attracted widespread attention in anion sensing because of their favorable photophysical and chemical properties and the ease of synthetic modification *via* ligands.^30–33^ Although Ru(ii) complexes have been reported to exhibit a luminescent “turn-on” response toward ReO_4_^−^, the reported detection system often relies on organic media and suffers from low sensitivity (∼0.1 mM) due to high background emission.^28^ Herein, we address this challenge by introducing a dipyridophenazine (dppz) ligand into Ru(ii) complexes to suppress background emission.^34,35^ Through rational design of the ligand microenvironment in Ru(ii)-dppz complexes, the presence of ReO_4_^−^ can induce molecular aggregation of the complex in a pure aqueous phase. In this system, the ReO_4_^−^-induced aggregation creates a local hydrophobic microenvironment for the hydrophobic dppz ligands, similar to DNA base pairs. This simultaneously triggers both the “light switch” effect and the AIE effect. Among a series of Ru(ii) polypyridine complexes with different ligand structures-[Ru(dip)_3_]^2+^ (Ru1), [Ru(dppz)_3_]^2+^ (Ru2), and [Ru(dip)_2_dppx]^2+^ (Ru3), where dip = 4,7-diphenyl-1,10-phenanthroline, dppz = dipyridophenazine, and dppx = 11,12-dimethyldipyridophenazine-the optimal complex, Ru3, achieves ultra-high sensitivity in a “turn-on” luminescent response to ReO_4_^−^ in pure aqueous medium, with LODs of 3.4 nM and 0.13 nM without and with preconcentration, respectively.

## 2 Experimental

### 2.1 Chemicals and materials

All reagents and materials in the experiment were analytical pure reagent grades obtained from commercial sources and were used without further purification. Perrhenate (ReO_4_^−^) and nitrate (NO_3_^−^) were purchased from Sinopharm Chemical Reagent Shaanxi Co., Ltd, bromine salt (Br^−^) was purchased from Tianjin Kemio Chemical Reagent Co., Ltd, nitrite (NO_2_^−^) was purchased from Beijing Tian you Fu kang Biotechnology Co., Ltd, chloride salt (Cl^−^), carbonate (CO_3_^2−^) and phosphate (PO_4_^3−^) were purchased from Tianjin Tianli Chemical Reagent Co., Ltd, bicarbonate (HCO_3_^−^) was purchased from Sigma Aldridge (Shanghai) Trading Co., Ltd, acetate (CH_3_COO^−^), biphosphate (HPO_4_^2−^) and sulfate (SO_4_^2−^) were all purchased from Sheng gong Bioengineering (Shanghai) Co., Ltd, Cleanert IC-Ag solid phase extraction column (F02865) was purchased from Agela Technologies, CNWBOND HC-C18 SPE Cartridge (SBEQ-CA0851) was purchased from CNW Technologies, and solutions were prepared using ultrapure water (18.2 M Ω) and used in all experiments.

### 2.2 Instrument

Ultrapure water was obtained from the German MERCKCLX-700Milli-Q filtration system with a resistivity of 18.2 M Ω. Luminescence spectroscopy test was measured on the Edingburgh FS5 fluorescence spectrometer (*λ*_ex_ = 450 nm, *λ*_ex_ = 650 nm), the slit was 2 nm, and spectral measurement was performed using a Q-204 standard covered fluorescent cuvette (1 cm × 1 cm), with a light path of 10 mm and a volume of 3.5 mL. Ultraviolet-visible (UV-VIS) absorption spectra were recorded on a T700 spectrophotometer and spectral measurements were performed using a Q-104 standard cover round bottom cuvette (1 cm × 1 cm) with a light path of 10 mm and a volume of 3.5 mL. The portable blue light transmitter consists of a dark cavity with a length of 12.5 cm, a width of 9.2 cm and a height of 7.4 cm, a blue light LED light strip (ex: 450 nm) and a power cord, and images are obtained through a smartphone. The molecular optical switch probe and its ^1^HNMR with ReO_4_^−^ were determined on a 600 MHz Avance Neo nuclear magnetic resonance spectrometer. Dynamic light scattering method (DLS) was used to characterize metal polypyridine Ru complex and its aqueous complex with perchlorate by MalvernNanoZS90. The metal polypyridine Ru complex was dispersed in ultrapure water and the DLS spectrum (10 readings per time) and the Zeta potential were recorded three times to was recorded three times (10 readings per time) to obtain the average. The AFM imaging was conducted using the scanning probe microscope manufactured (Japan Seiko SPI 3800N SPA-400).

### 2.3 Luminescence and absorption measurements

Add Ru(ii) complexes (20 µM) to a solution containing different concentrations of ReO_4_^−^ or 10 µM of other ions or mixed ions (10 µM), and then add ultrapure water to 2 mL in a 3.5 mL quartz cuvette for luminescence measurement. The luminescence spectra were recorded at room temperature using an excitation wavelength of 450 nm, an emission range of 550–800 nm and a slit width of 2 nm, respectively. The luminescence intensity at 650 nm were used to plot the calibration curves for ReO_4_^−^.

A similar procedure was employed for monitoring the absorbance changes of Ru(ii) complexes solution (40 µM) caused by the analytes.

### 2.4 Particles size, zeta potential and TEM measurements and atomic force microscopy (AFM) images

For particle size measurement, Ru(ii) complexes (10 µM) was dispersed in ultrapure water and DLS spectrum was recorded thrice (10 runs in each reading) to consider the average value. Similarly, the average of three readings was also considered for measurements of Ru(ii) complexes with different concentrations of ReO_4_^−^ (5 µM, 10 µM and 20 µM). For determining the net charge, the zeta potential was recorded thrice for each sample and average was considered. The aqueous solution of the molecular probe and ReO_4_^−^ composite was TEM-characterized by using a H-7650 (Hitachi) instrument at 200 kV. The solution droplets were placed on a carbon coated copper grid (3 mm diameter) meter and placed at room temperature for about 5 min. Ru3 solutions without ReO_4_^−^ and with 10 µM ReO_4_^−^ were prepared respectively. 10 µL volume of each solution was deposited on a glass slide, followed by drying at room temperature prior to AFM characterization.

### 2.5 Lifetime measurements

Lifetime experiments were performed to monitor the luminescence lifetime decay of Ru(ii) complexes in the absence and presence of ReO_4_^−^ at 405 nm pulse laser excitation (FluoTime 100, PicoQuant).

### 2.6 Detection of rhenium in simulated hanford waste solution

To test the ability of the Ru3 to detect ReO_4_^−^ from groundwater, the simulated Hanford low-activity waste was prepared. The major inorganic constituents present in the groundwater are listed in Table S3. After diluting the simulated water sample 1000-fold, ReO_4_^−^ at different concentrations from 0.5 to 10 µM was added for titration experiments.

### 2.7 Sample pretreatment and IC-Ag column regeneration

The 20 mL water samples in the presence and absence of 1.5 nM ReO_4_^−^ were first treated by a Cleanert IC-Ag column, then placed in a 100 mL beaker, heated and evaporated to the solution volume were <200 µL in the microwave oven for 10 min at mid-high level, then transferred to the centrifuge tubes and filled to 200 µL with ultrapure water, and then used for luminescence measurement.

After each use, the IC-Ag column was regenerated following the procedure. The column was washed twice with 10 mL of 1 : 1 NH_4_OH and then rinsed with deionized water 4–5 times. The column was then washed twice with 10 mL of 1 : 1 HNO_3_ and finally rinsed with deionized water until the effluent reached neutral pH. The column was then re-loaded with 5% AgNO_3_ solution to restore its capacity for subsequent use.

### 2.8 Synthesis and characterization

Ru(ii) complex references were synthesized^36–38^ and characterized by nuclear magnetic and mass spectrometry. The results are as follows.

#### 2.8.1. [Ru(dip)_3_]Cl2 (Ru1)

Yield: 60%. ^1^H NMR (400 MHz, CD_3_OD) *δ* 8.43 (d, *J* = 5.3 Hz, 6H), 8.36 (s, 6H), 7.81 (d, *J* = 5.5 Hz, 6H), 7.75–7.60 (m, 30H). ESI-MS [M–2Cl]^2+^ calculated for C_72_H_48_N_6_Ru 549.1440; found 549.1492.

#### 2.8.2. [Ru(dppz)_3_]Cl_2_ (Ru2)

Yield: 50%. ^1^H NMR (400 MHz, CD_3_OD) *δ* 9.81 (dd, *J* = 8.3, 1.2 Hz, 18H), 8.51 (dt, *J* = 6.8, 3.4 Hz, 18H), 8.44 (dd, *J* = 5.5, 1.3 Hz, 18H), 8.15 (dt, *J* = 6.6, 3.4 Hz, 18H), 7.94 (dd, *J* = 8.3, 5.4 Hz, 18H). ESI-MS [M–2Cl]^2+^ calcd for C_54_H_30_N_12_Ru 474.0880; found 473.9076.

#### 2.8.3. [Ru(dip)_2_dppx]Cl_2_ (Ru3)

Yield: 75%. ^1^H NMR (400 MHz, CD_3_OD) *δ* 9.84 (d, *J* = 8.3 Hz, 2H), 8.47 (d, *J* = 5.6 Hz, 2H), 8.45–8.34 (m, 8H), 8.31 (s, 2H), 8.00 (dd, *J* = 8.2, 5.4 Hz, 2H), 7.80 (dd, *J* = 10.3, 5.5 Hz, 4H), 7.75–7.58 (m, 20H), 2.74 (s, 6H). ESI-MS [M–2Cl]^2+^ calculated for C_68_H_46_N_8_Ru 538.1445; found 537.9564.

### 2.9 Single crystal culture and analysis

[Ru(dip)_2_dppx]^2+^/ReO_4_^−^ Complex: excess ReO_4_^−^ aqueous solution was added to [Ru(dip)_2_dppx]^2+^ aqueous solutions, respectively, to generate precipitation, take the supernatant, then dissolve the precipitate in acetonitrile solution, then take 0.6 mL of the solution into a 2 mL glass vial, then add 0.4 mL of ultrapure water, mix evenly, follow-up operations are the same as above, obtain orange-red single crystal. Crystals were tested on a Bruker D8 VENTURE TXS PHOTON II diffractometer. During the data collection process, MoKα light source (*λ* = 0.71073) was used for radiation, and the crystal temperature was maintained at 193.00 K. A total of 216 903 reflections were measured (3.906°<2*θ* ≤ 49.424°). The structure is analyzed by the SHELXT^39^ structure using the inherent phase method, and the least squares minimization method is used to refine it through the SHELXL^40^ optimization package of OLEX2 (ref. 41). A total of 36 784 independent reflections were used for all calculations. The CCDC 2501068 contains the crystallographic data for the crystal structure in this paper.

### 2.10 DFT theoretical calculations

All calculations were carried out with the Gaussian 16 A.03 software.^42^ The PBE0 (ref. 43) functional was adopted for all calculations in combination with the Grimme's D3(BJ)^44,45^ dispersion correction. The H atoms were optimized with def2-SVP.^46,47^ basis set was used for all atoms. The Gaussian 16 A.03 (ref. 42) software was invoked by sobEDA^48^/Multiwfn 3.8(dev)^49,50^ software to perform the energy decomposition calculation at the PBE0-D3(BJ)/def2-TZVP calculation level. The structural figures were rendered by means of the VMD 1.9.3 (ref. 51) visualization program.

## 3 Results and discussion

### 3.1 Feasibility validation of the proposed turn-on AIE sensor for ReO_4_^−^ detection

**Fig. 1:**
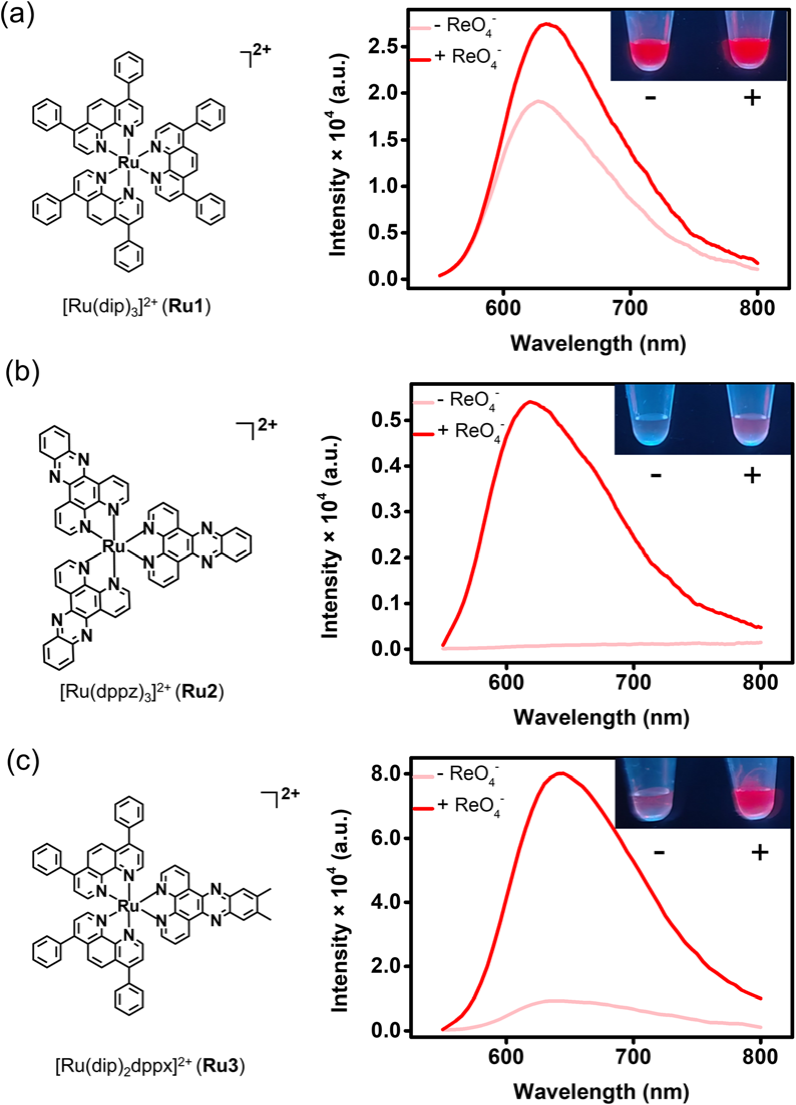
Feasibility validation of the proposed turn-on AIE sensor for ReO_4_^−^ detection in aqueous media. (a–c) The effect of ligand structure of Ru1 (a), Ru2 (b), Ru3 (c) complexes on luminescence response to ReO_4_^−^ anion. Left: chemical structures; right: luminescence spectra upon addition of ReO_4_^−^. The inset shows the corresponding change in colour of the Ru1–Ru3 complex solutions before and after addition of ReO_4_^−^ under 450 nm blue light.

To understand the effect of ligand structure of Ru(ii) complex on ReO_4_^−^ anion binding, Ru1, Ru2 and Ru3 were prepared and characterized by ^1^H NMR and mass spectrometry (Fig. S1–S3). Their luminescence signal responses were studied in the presence of ReO_4_^−^ (Fig. 1a–c). The results show that with the increase of ReO_4_^−^ anion concentration, only Ru2 and Ru3 showed strong luminescence emission enhancement. Compared with the Ru1 containing only the dip ligand and the Ru2 containing only the dppz ligand, the sensitivity of the Ru3 is significantly improved due to combining the hydrophobicity of the auxiliary ligand dip and the “light-switch” characteristics of dppx ligand.^35,52^

### 3.2 Luminescence sensing performance of Ru3 for ReO_4_^−^

**Fig. 2:**
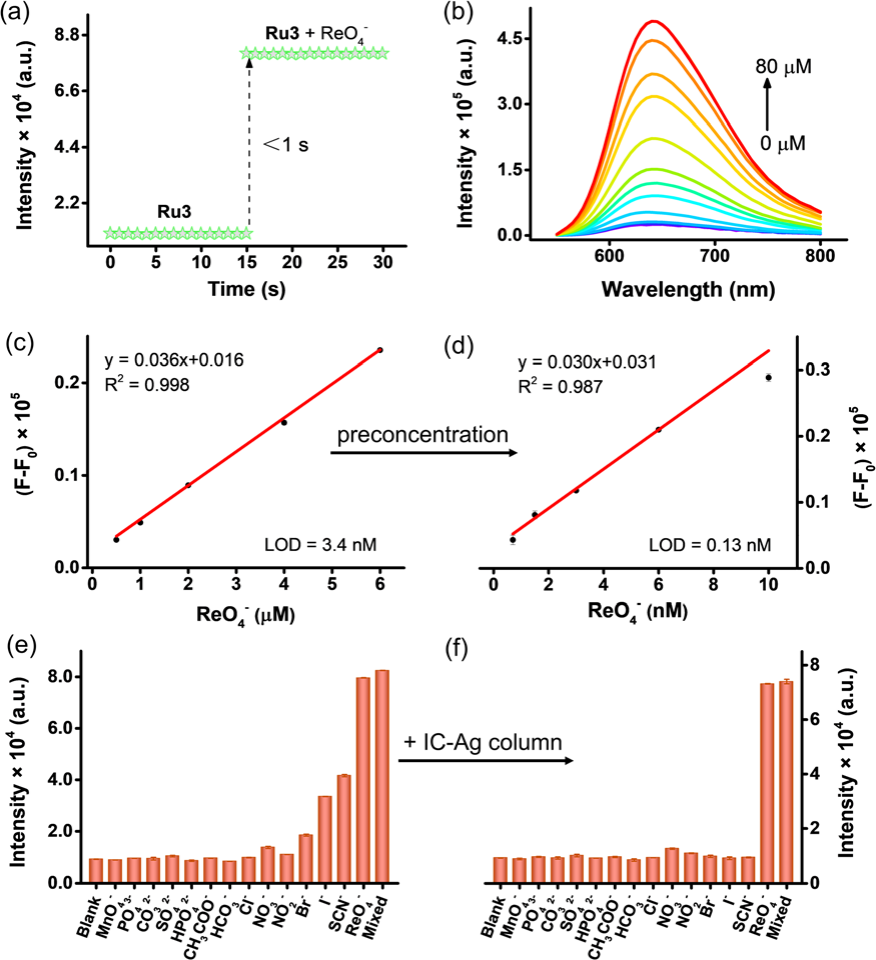
Luminescence sensing performance of Ru3 for ReO_4_^−^ in aqueous solution. (a) Emission spectra of Ru3 upon addition of ReO_4_^−^. (b) Emission intensity of Ru3 aqueous solution recorded at different time intervals with and without ReO_4_^−^ (10 µM). (c and d) Linear relationship between the change in luminescence intensity (*F*–*F*_0_, where *F* and *F*_0_ are the intensities after and before adding ReO_4_^−^, respectively) and the concentration ReO_4_^−^ without (c) and with (d) preconcentration. (e and f) Selectivity of Ru3 toward ReO_4_^−^ over other anions (10 µM) without (e) or with (f) an IC-Ag column. Error bars represent the standard deviation of three independent measurements.

By observing the time-dependent response process of ReO_4_^−^, it was found that the Ru3 responded immediately after adding ReO_4_^−^, with a response time of <1 second (Fig. 2a). Further, stability experiments of Ru3 reveal that the luminescence response of the Ru3 remained almost unaltered even after 7 days, indicating their excellent stability in the room temperature (Fig. S4). Therefore, Ru3 can be used as a molecular “light-switch” probe to monitor of ReO_4_^−^ in water sensitively, rapidly and stably.

Luminescence titration experiment is also carried out by gradual addition of ReO_4_^−^ anion to the aqueous solution of Ru3 (20 µM). Upon addition of increasing amount of ReO_4_^−^ anion, gradual increases in Luminescence intensity (Fig. 2b) at 630 nm are observed. The Job plot analysis shows the inflection point at 0.67 indicating the 1 : 2 stoichiometry between the probe and anion (Fig. S5). Further through luminescence spectral titration data, there is a good linear relationship between the luminescence emission intensity and the low concentration of ReO_4_^−^ at 630 nm. The limit of detection (LOD) of ReO_4_^−^ for Ru3 calculated using 3σ/*k* (where *σ* is the standard deviation of the blank solution and *k* is the slope of the fitted line) was 3.4 nM (Fig. 2c), which is two orders of magnitude higher than the recently reported turn-on probes (Table S1). One notable exception is a study employing a solid-phase extraction approach with functionalized glass microspheres, which achieved a sensitivity of 0.26 nM.^26^ Upon applying evaporation-assisted pre-concentration,^53^ the LOD of Ru3 for ReO_4_^−^ can be lowered to 0.13 nM (Fig. 2d and S6), thereby meeting the WHO guideline of 1.6 nM.^54^ To the best of our knowledge, this represents the most sensitive method reported to date.

In practical applications, the selectivity of luminescence sensors for specific analytes is crucial. The luminescence response of Ru3 in the presence of 13 different anions (SCN^−^, I^−^, Br^−^, NO_2_^−^, NO_3_^−^, Cl^−^, HCO_3_^−^, CH_3_COO^−^, HPO_4_^2−^, SO_4_^2−^, CO_3_^2−^, PO_4_^3−^, MnO_4_^−^), cations (Na^2+^, Mg^2+^, Fe^2+^, Cu^2+^, K^+^, Ca^2+^,UO_2_^2+^), and organic compounds (SDS and SDBS) that are common in the environment was tested. The results show that compared with ReO_4_^−^, except for I^−^, SCN^−^ and Br^−^, other anions and cations only cause weak luminescence enhancement or even no response (Fig. 2e and S7). However, the interference ions I^−^, SCN^−^, Br^−^ and organic compounds SDS and SDBS can be effectively removed by sample pretreatment using IC-Ag columns (Ag^+^ with I^−^, SCN^−^, Br^−^ anions to form sparingly soluble silver salt precipitates) and HC-C18 cartridges (Fig. 2f and S8–9). In addition, even in the presence of mixed anions (13 different anions), Ru3 still exhibited an excellent luminescence response to ReO_4_^−^ after adding 10 µM ReO_4_^−^ (Fig. 2e and f), indicating that Ru3 can be used as a selective “light-switch” probe and can also be used for the identification and detection of ReO_4_^−^ in complex ambient water samples.

### 3.3 Response in the simulated waste and tap water samples

**Fig. 3:**
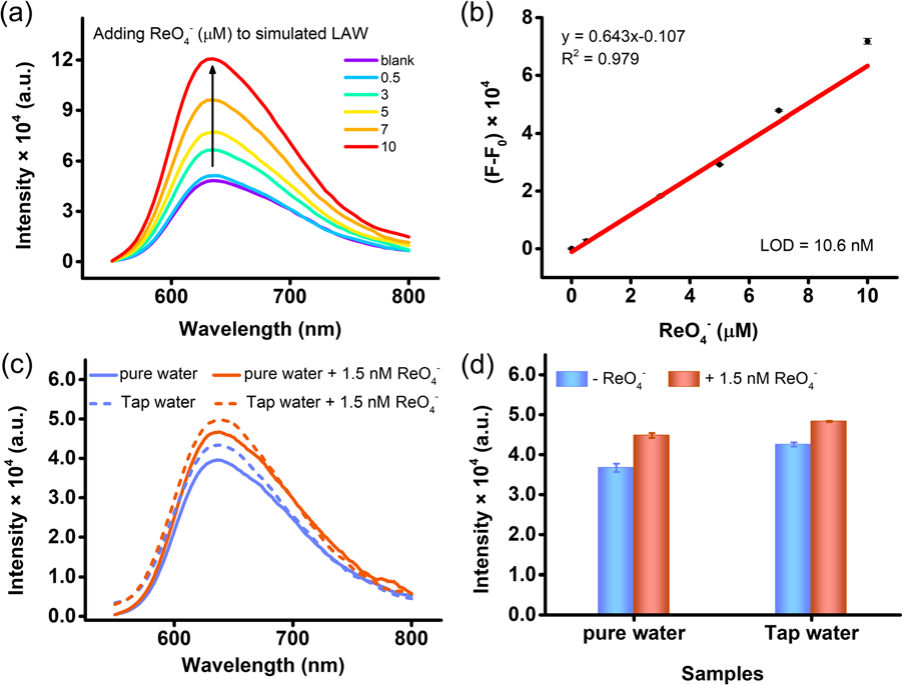
Determination of ReO_4_^−^ in real samples using Ru3. (a) Emission spectra of Ru3 with different concentrations of ReO_4_^−^ in simulated LAW. (b) Linear correlation between the concentration of ReO_4_^−^ concentration and the increase in luminescence intensity in simulated LAW. (c) Emission spectra and (d) the corresponding intensity at *λ*_em_ = 650 nm for Ru3 after preconcentration of different water samples.

To verify that Ru3 could detect TcO_4_^−^ in Hanford low-activity waste (LAW) solution, which contains high concentration of other ions and low level of TcO_4_^−^ (Table S2),^55,56^ we further investigated the response of Ru3 in the simulated waste solution and the results have been presented in Fig. 3a and b. Our results show a linear response towards ReO_4_^−^ with a LOD of 10.6 nM. Meanwhile, through the pretreatment of tap water samples, the detection of 1.5 nM ReO_4_^−^ in water was achieved (Fig. 3c and d), which is below the World Health Organization's guidance level (100 Bq L^−1^ = 1.61 nM).^54^ Therefore, Ru3 can serve as an efficient fluorescent sensing probe for detecting low-concentration ReO_4_^−^/TcO_4_^−^ pollution.

### 3.4 Mechanistic studies

**Fig. 4:**
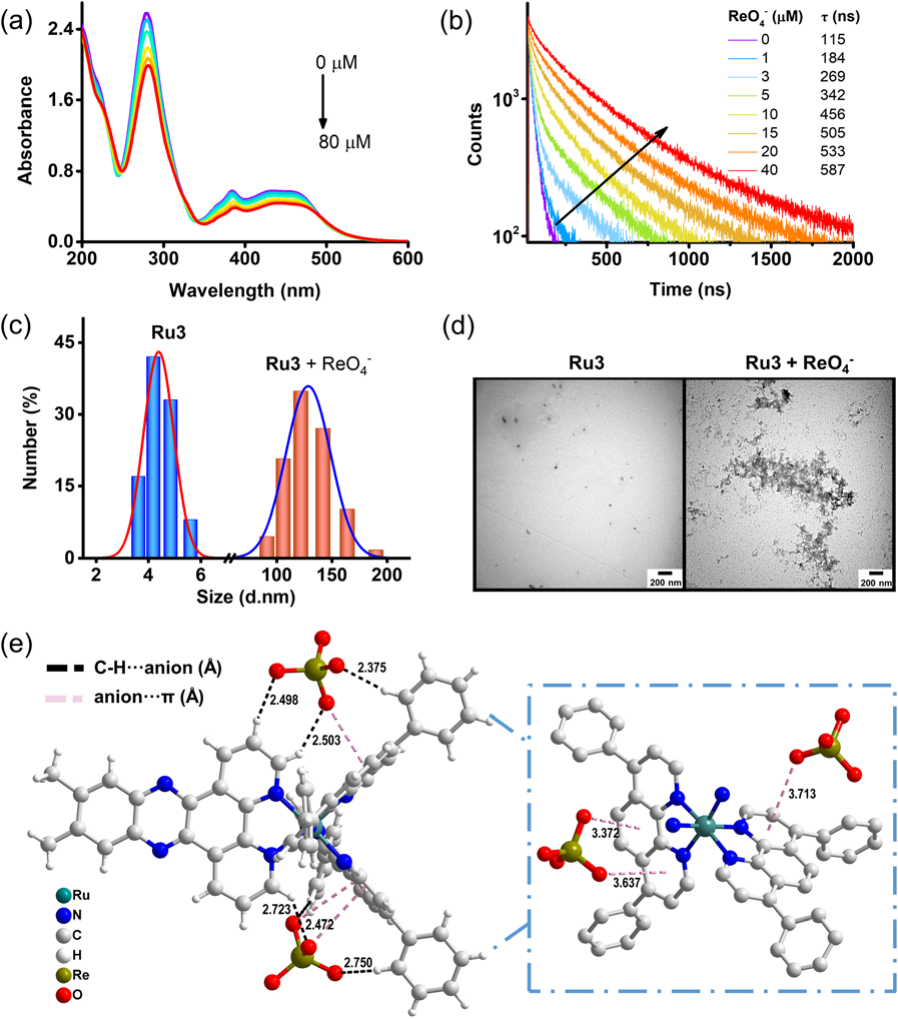
Sensing mechanism of Ru3 for recognition of ReO_4_^−^. (a) UV-Vis absorption spectra of Ru3 with different concentrations of ReO_4_^−^. (b) Time-resolved luminescence decay curves of Ru3 with different concentrations of ReO_4_^−^. The corresponding lifetimes (*τ*) are provided in the legend. Comparative DLS (c) and TEM (d) studies of Ru3 in the absence and presence of ReO_4_^−^. (e) Crystal structure of [Ru3](ReO_4_)_2_ adduct.^59^ Black and pink dotted line represents the C–H⋯anion and the anion⋯π interactions between cations and anions, respectively. The region highlighted by the blue dashed box provides a close-up view of the anion⋯π interaction.

To clarify the potential sensing mechanism, the complexation process of the probe and ReO_4_^−^ was studied through UV-vis absorption spectrum, lifetime studies, zeta potential measurement, particle size analysis and TEM images. The UV spectral titration data showed that the absorption of Ru3 around 383 nm (the dppx chromophore) upon addition of ReO_4_^−^ decreased gradually (Fig. 4a). This behaviour is consistent with that of classical DNA-binding light-switch complexes, indicating that the dppx ligand of Ru3 is also situated within a hydrophobic environment, as is the case during its intercalation into DNA.^35,57,58^ In addition, time-resolved measurements with ReO_4_^−^ in water shows that the lifetime of Ru3 is positively correlated with the ReO_4_^−^, and it gradually increases from 115 ns to 587 ns (Fig. 4b). The extended probe lifetime may be attributed to the ReO_4_^−^ anion inducing Ru3 self-aggregation and protect the dppx ligand from contacting with water molecules, thereby reducing non-radiative decay.

The positively charged Ru3 with hydrophobic properties exhibits a strong tendency to bind to weakly hydrated ReO_4_^−^ anions through electrostatic and hydrophobic effects. After the addition of ReO_4_^−^, the net charge of Ru3 decreased from +15.30 mV to +7.54 mV (Fig. S10), indicating that cationic Ru3 showed strong binding affinity for negatively charged ReO_4_^−^.

DLS spectroscopy showed the size distribution of aggregates induced by ion interaction in the solution, further supporting the formation of probe/ReO_4_^−^ complexes, because after the addition of ReO_4_^−^, Ru3 showed mean fluid dynamic diameters of 135.3 nm, while in the absence of ReO_4_^−^, the average particle sizes of the aqueous solutions of Ru3 were 4.4 nm (Fig. 4c). Similarly, TEM measurements supported the conclusion of aggregate formation. The aqueous solutions with [Ru3]Cl_2_ alone showed smaller dispersed particles, which formed larger aggregates upon addition of ReO_4_^−^ (Fig. 4d). Concentration-dependent DLS studies showed that the average hydrodynamic diameter of the complex increased progressively with increasing ReO_4_^−^ concentration (Fig. S11). AFM characterization (Fig. S12) corroborated the DLS and TEM data, providing visual evidence that ReO_4_^−^ triggered the self-assembly and aggregation of the ruthenium(ii) complex. Therefore, it can be clearly seen from the above results that the Ru3 will experience strong self-aggregation after binding to ReO_4_^−^, and the ReO_4_^−^-induced aggregation creates a local hydrophobic microenvironment for the hydrophobic dppz ligands, similar to DNA base pairs. This simultaneously triggers both the “light switch” effect and the AIE effect, thereby achieving monitoring of the luminescence turn-on of ReO_4_^−^.

In order to further identify and analyse the weak interaction between Ru3 and ReO_4_^−^ in detail, the single crystal analysis of Ru3 and ReO_4_^−^ complex was carried out (Table S3). As shown in the Fig. 4e, the complex is crystallized in monoclinic system and belongs to *P*2_1_/*c* space group. The Ru atom adopts a distorted octahedral geometry, in which four coordination sites are occupied by the N atom on the dip ligand, and the remaining two coordination sites are occupied by the N atom on the dppx ligand. Crystallographic details, bond angles and bond lengths are included in the supplementary data. In addition, the solid structure of the complex reveals the existence of C–H⋯O hydrogen bond interaction (2.375–2.750 Å) between Ru3 and ReO_4_^−^ anion. At the same time, the single crystal results show that there is an anion⋯π interaction (3.372–3.713 Å) between the O atom of ReO_4_^−^ and the dip ligand of Ru3. The crystal stacking shows that the anion ReO_4_^−^ plays a vital role in the formation of 3D stacking (Fig. S13). Therefore, in the case of ReO_4_^−^, the formation of 3D stacking network may help Ru3 to form aggregates in solid or solution state.

Further structural optimization and energy decomposition calculations were performed on [Ru3](ReO_4_)_2_ using Gaussian16 *via* density functional theory (DFT) (Fig. S14). The results indicate that the total interaction energy Δ*E*_(int) between the ReO_4_^−^ anion and Ru3^2+^ represents the total energy released upon ion-pair formation ([Ru3](ReO_4_)_2_: −221.28 kcal mol^−1^), demonstrating that this binding is thermodynamically favorable. Concurrently, the electrostatic interaction energy (Δ*E*_els) was −213.51 kcal mol^−1^, indicating that strong electrostatic attraction is the primary driving force for binding. Furthermore, Δ*E*_repulsion and Δ*E*_dispression terms from hydrophobic interactions constitute the important portion of the binding energy, underscoring the importance of hydrophobic effects in the sensitive detection of ReO_4_^−^. These results collectively provide theoretical evidence that the ReO_4_^−^ light-switching mechanism involves the synergistic effects of electrostatic and hydrophobic interactions.

## 4 Conclusion

In summary, this work successfully developed a novel ruthenium(ii) dipyridophenazine complex, [Ru(dip)_2_dppx]^2+^ (Ru3), as a highly sensitive and selective turn on luminescent probe for detecting perrhenate (ReO_4_^−^) in aqueous media. The proposed sensing mechanism involves the synergistic effect of the light switching effect and the AIE effect, driven by anion exchange, and achieved through electrostatic attraction, anion⋯π, C–H⋯anion hydrogen bonding and π–π stacking interactions. The probe integrated with preconcentration exhibits a detection limit of 0.13 nM, establishing it as one of the most sensitive methods reported to date. Its practical utility was demonstrated through the accurate determination of ReO_4_^−^ in complex matrices. This provides a new molecular platform with excellent performance and significantly enhanced practicality for addressing the challenge of environmental monitoring of radioactive TcO_4_^−^. Beyond demonstrating high sensitivity, selectivity (13 different anions) and rapid response (within 1 s), this study also introduces a generalizable design strategy for anion sensing based on ruthenium(ii) dppz complex.

## Author contributions

Yanni Li: conceptualization, investigation, experimental operation, data analysis and mechanism verification, and writing – original draft preparation and revision. Yanxin Du and Hao Liu: investigation, experimental operation, data analysis. Yijie Zhu and Fan Deng: experimental operation, data analysis. Qinfeng Xu: supervision and guidance, writing review and revision, project administration, funding acquisition. All authors have agreed to the final version of the manuscript.

## Conflicts of interest

There are no conflicts to declare.

## Supplementary Material

RA-016-D6RA01192F-s001

RA-016-D6RA01192F-s002

## Data Availability

CCDC 2501068 (for [Ru3](ReO_4_)_2_) contains the supplementary crystallographic data for this paper.^59^ All data supporting the findings of this study are available within the article and its supplementary information (SI). Supplementary information: additional ^1^H NMR spectra of all synthesized complexes, luminescence spectra, zeta potential analysis, dynamic light scattering (DLS) profiles, atomic force microscopy (AFM) images, theoretical calculation data, and detailed crystallographic information. See DOI: https://doi.org/10.1039/d6ra01192f.
