# Supplementary material for: A turn-on AIE sensor for nanomolar detection of perrhenate in aqueous media
Source: RSC Adv. 2026 Mar 26;16(19):16759–66. doi: 10.1039/d6ra01192f (PMC13019534; doi:10.1039/d6ra01192f)
Supplement: RA-016-D6RA01192F-s001 [file RA-016-D6RA01192F-s001.pdf]

Supplementary Information (SI) for RSC Advances.
This journal is © The Royal Society of Chemistry 2026

Supporting Information

A Turn-On AIE Sensor for Nanomolar Detection of Perrhenate in Aqueous Media

Yan-ni Li, Yan-xin Du, Hao Liu, Yi-jie Zhu, Fan Deng, Qin-feng Xu*

School of Food Science and Engineering, National Research and Development Center for Goat Dairy Products Processing Technology, Shaanxi University of Science and Technology, Xi'an, Shaanxi 710021, China.

* Corresponding Authors. Email: xuqinfeng@sust.edu.cn.

1. Supplementary Figures

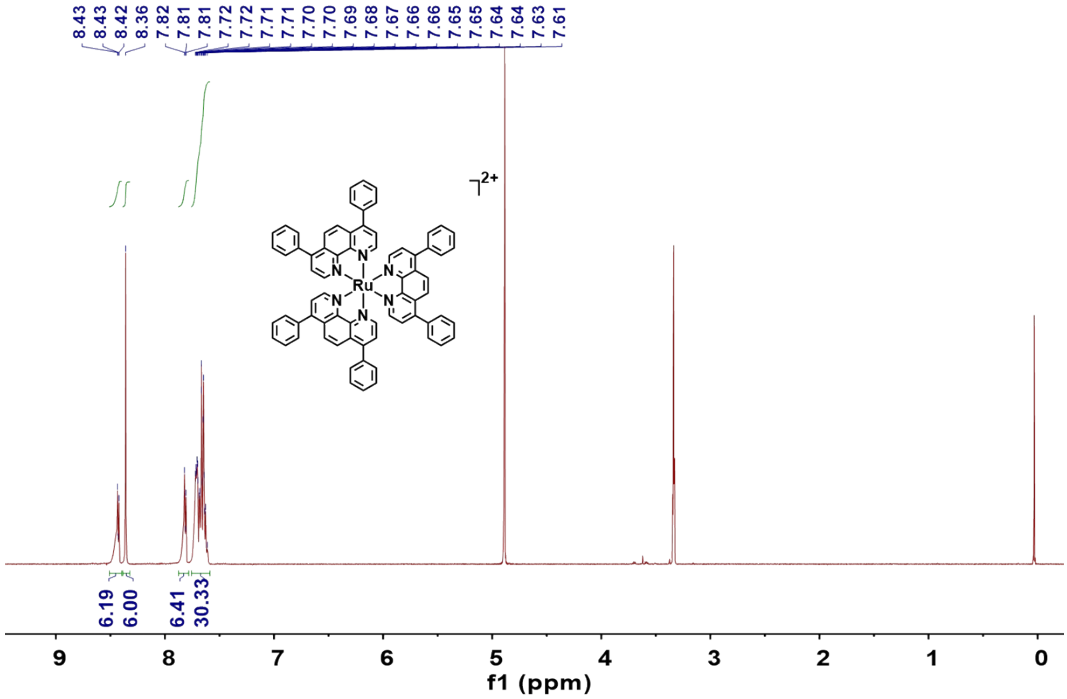

Fig. S1 ^{1}H NMR spectrum of probe **Ru1** in CD_4O.

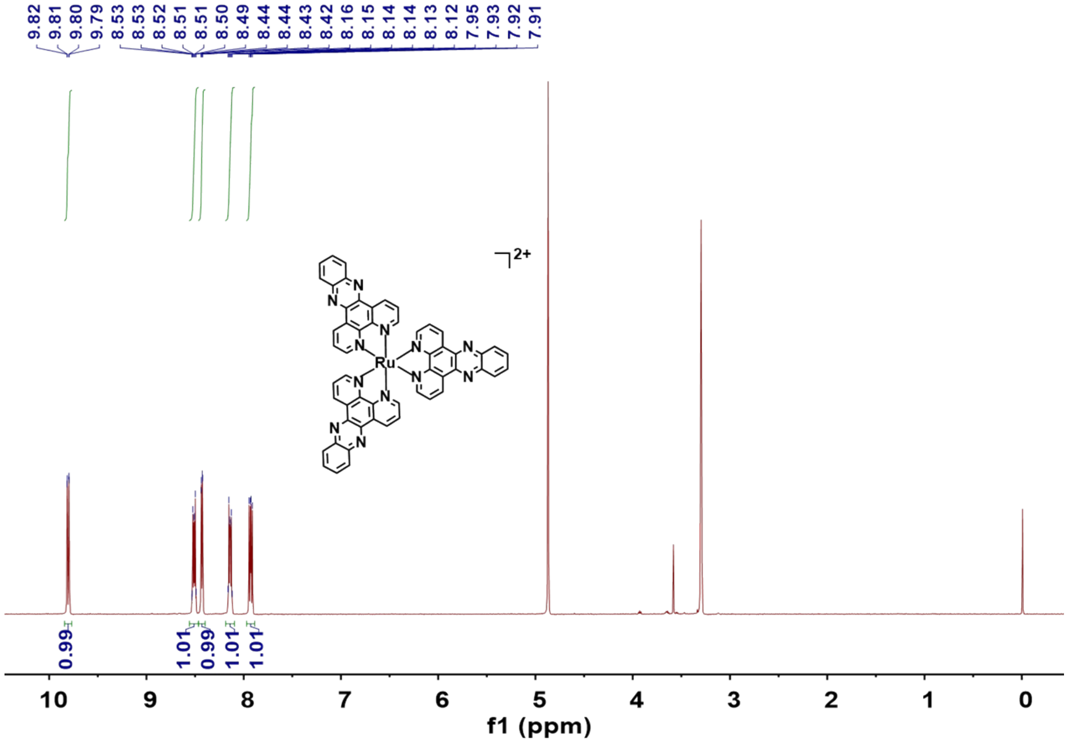

Fig. S2 ^{1}H NMR spectrum of probe **Ru2** in CD_4O.

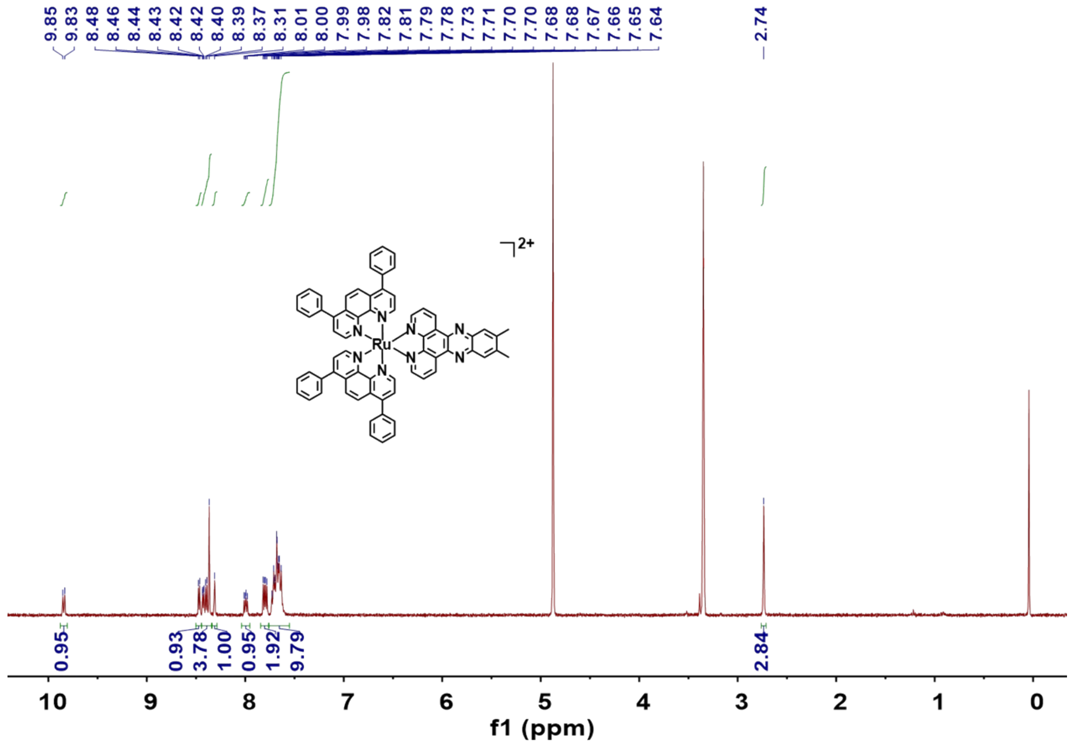

Fig. S3 ^{1}H NMR spectrum of probe **Ru3** in CD_4O.

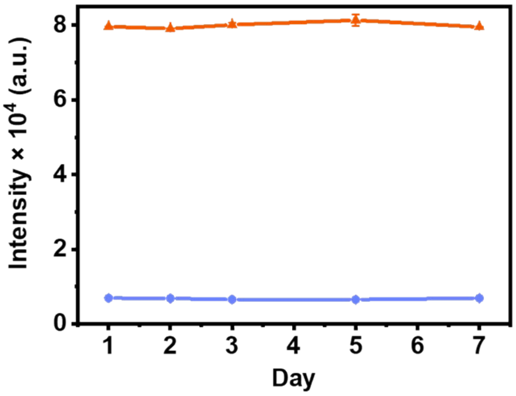

Fig. S4 Stability test of **Ru3** aqueous solution at room temperature over 7 days in the absence and presence of ReO_4^-.

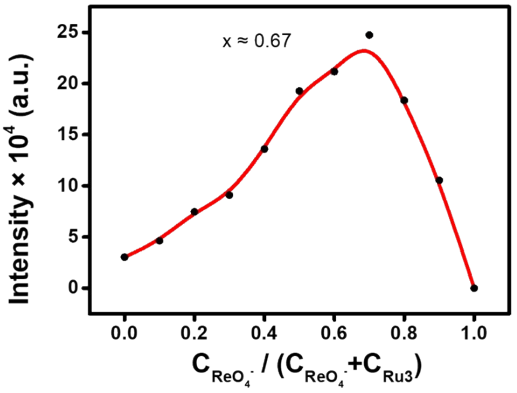

Fig. S5 Job plot of Luminescence intensity of **Ru3** with ReO_4^- in water (total concentration: 50 μM).

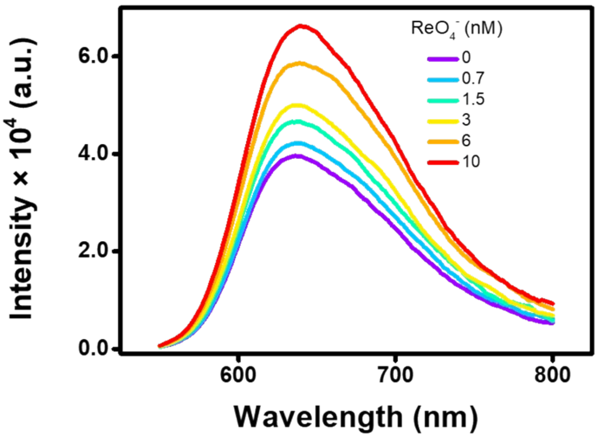

Fig. S6 Luminescence emission spectra of **Ru3** in the presence of different concentrations of ReO_4^- with preconcentration.

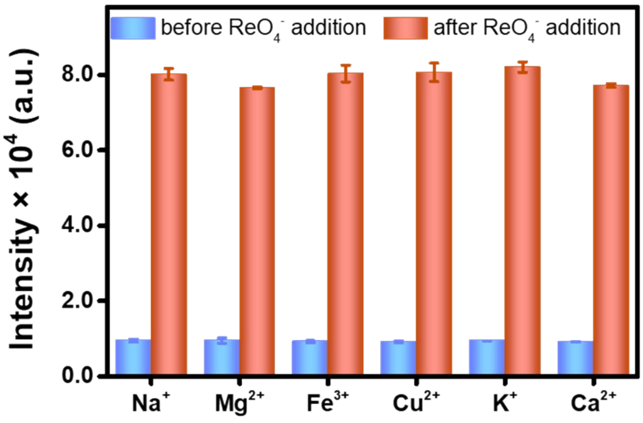

Fig. S7 Luminescence responses of probe **Ru3** in the presence of different cationic analytes.

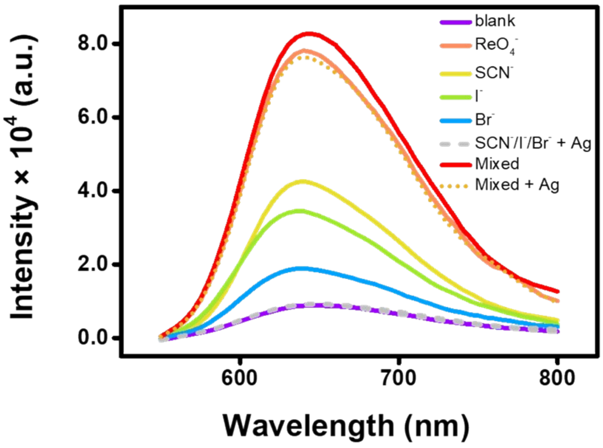

Fig. S8 Luminescence emission spectra of **Ru3** to ReO_4^- and other anions without and with treatment of IC-Ag column.

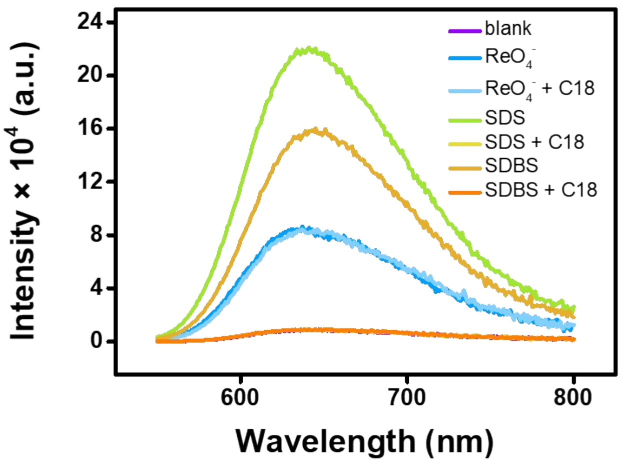

Fig. S9 Luminescence responses of **Ru3** (20 μM) toward ReO_4^- and other organic compounds (10 μM) without and with treatment of C18 column.

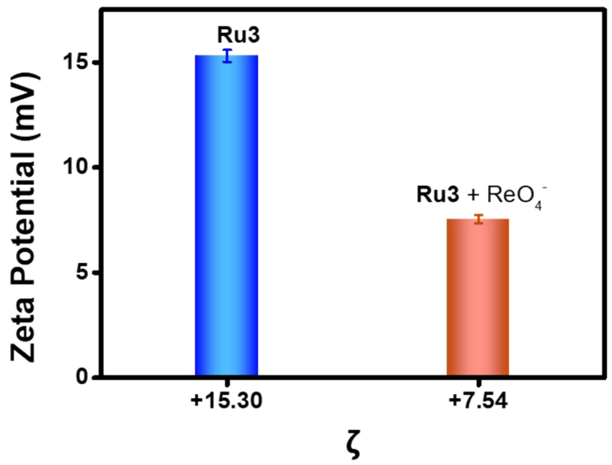

Fig. S10 Comparative Zeta potential study of **Ru3** in the absence and presence of ReO_4^-.

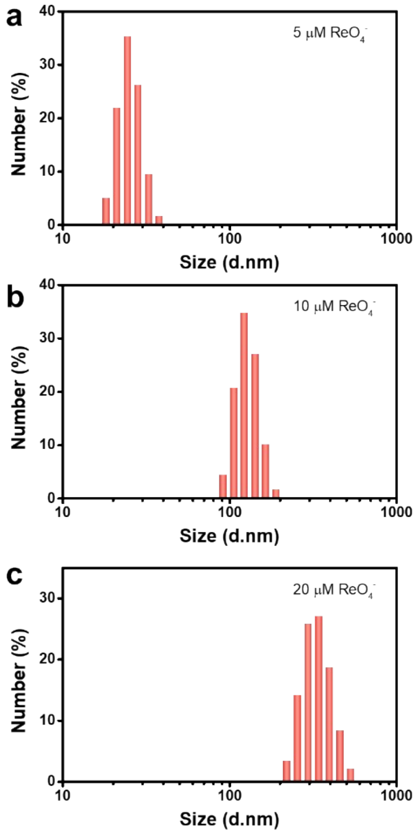

Fig. S11 DLS profiles of **Ru3** in the presence of different concentrations of ReO_4^-. (a) 5 μM，(b) 10 μM, (c) 20 μM.

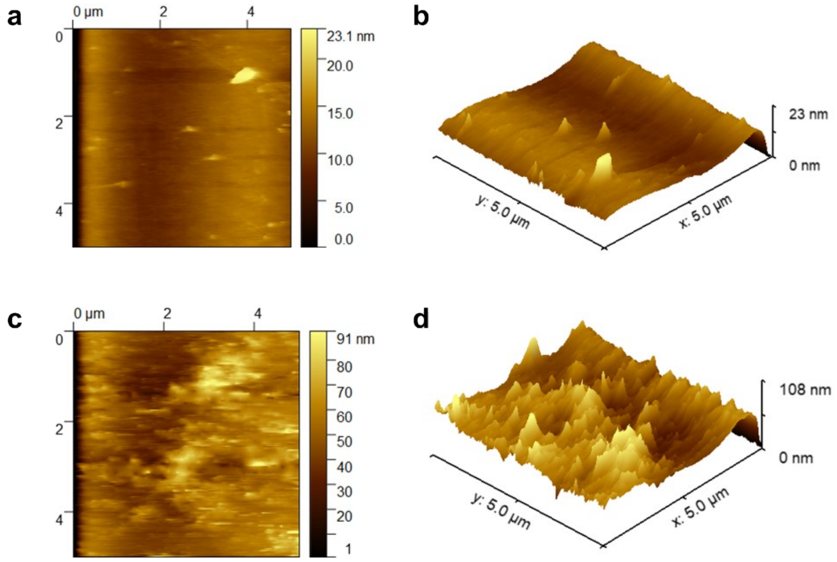

Fig. S12 AFM images of **Ru3** in the absence and presence of ReO_4^-. (a) 2D and (b) 3D image without ReO_4^-, (c) 2D and (d) 3D image with ReO_4^-.

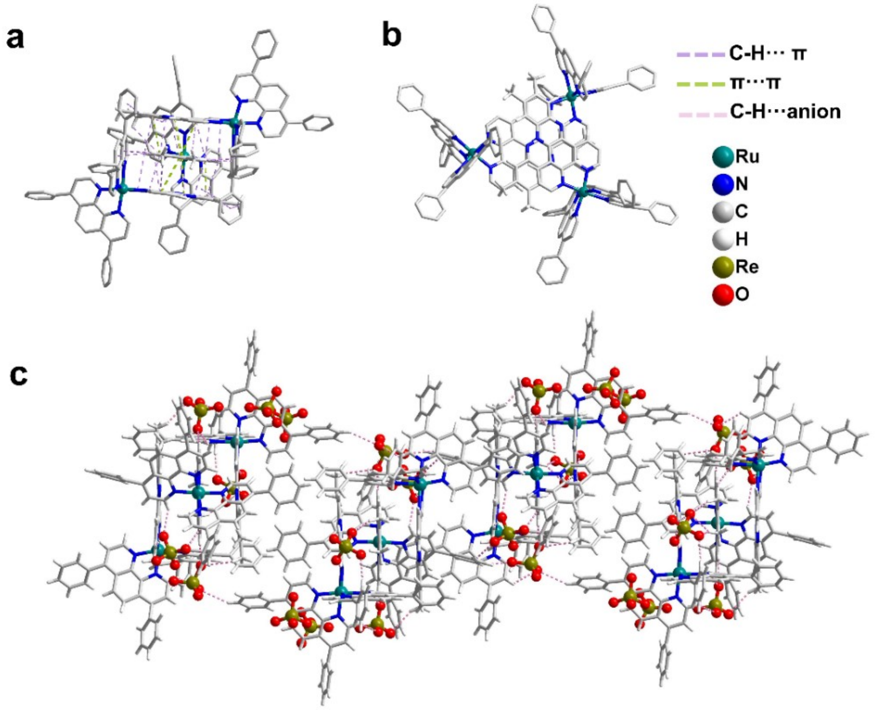

Fig. S13 (a) Side view and (b) top view of the crystal structure of [**Ru3**](ReO_4)$_2$ adduct complexes. Solvent molecules and hydrogen atoms are omitted for clarity. The purple and the green dotted line represent the C-H··· π interaction and the π···π interaction in the adjacent molecules of **Ru3**, respectively; (c) View of the three-dimensional (3D) crystal packing of [**Ru3**](ReO_4)$_2$ adduct complexes.

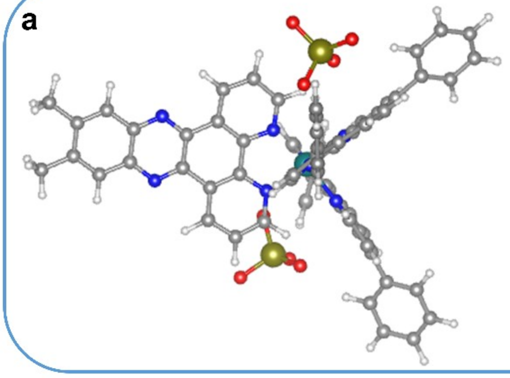

b

$E_{binding}$(kcal/mol)	[Ru3](ReO_4)$_2$
ΔE_int	-221.28
ΔE_els	-213.51
ΔE_repulsion	80.61
ΔE_orb	-63.48
ΔE_dispresion	-24.92

Fig. S14 (a) The optimized structure of the [**Ru3**](ReO_4)$_2$ complex. (b) Energy decomposition analysis of the [**Ru3**](ReO_4)$_2$ complex.

2. Supplementary Tables

Table S1. Sensitivity comparation of the present probe with the recently reported luminescence probes for ReO_4^-.

Detection manner	Materials	Detection mechanism	Solvent	Response time	LOD (μM)	Ref.
turn-off	CP-16	aggregation-induced fluorescence quenching	water and a chloroform/methanol (1:1) mixture	-	0.58	[1]
turn-off	TJNU-302	electron transfer	H_2O	-	90	[2]
turn-off	Zr^{4+} MOFs	electron transfer	H_2O		0.55	[3]
turn-off	NCU-2	ion exchange	H_2O	＜30 s	0.067	[4]
turn-off	PMA	Photoinduced electron transfer (PET)	H_2O	-	14	[5]
turn-off	MOCNs	PET	H_2O	-	12.6	[6]
turn-off	BTTA–BDNP	PET	DMF/H_2O	＜30 s	0.067	[7]
turn-off	TFPM-EP-Br	PET	H_2O	＜2 s	0.033	[8]
turn-off	TpBDOH-AB	ion exchange	C_2H_5OH	2 s	1.07	[9]
turn-off	C-CD	PET	H_2O	-	87	[10]
turn-off	$1H_6^{6+}$	PET	0.1 M CF_3SO_3Na, pH = 2	-	-	[11]
turn-off	ModA	hydrogen bond	20 mM Tris, 50 mM NaCl, pH = 7.6	-	-	[12]
turn-on	TPDC-I	AIE	H_2O	-	0.9	[13]
turn-on	XB	halogen bonding and electrostatic interaction	10 mM aqueous hepes buffer, pH = 7.4	-	-	[14]
turn-on	PIC	J-aggregation	H_2O	-	0.17	[15]
turn-on	ThT	AIE	H_2O	-	260	[16]
turn-on	AuO	J-aggregation	H_2O	-	270	[17]

Table S1: (continued)

Detection manner	Materials	Detection mechanism	Solvent	Response time	LOD (μM)	Ref.
turn-on	[Pt(tpy)Br](SbF_6)	ion exchange	H_2O	2-5 min	2.6×10^{-4}	[18]
turn-on	Ir-PAF	ion exchange	pH = 4	-	2.07	[19]
turn-on	Ru(II) complex	halogen-bond	acetonitrile	-	-	[20]
turn-on	**Ru1**	AIE	H_2O	<1 s	0.718	
turn-on	**Ru2**	AIE	H_2O	<1 s	0.026	
turn-on	**Ru3**	AIE	H_2O	<1 s	0.003	this work
turn-on	**Ru3**	AIE	H_2O	<1 s	1.3×10^{-4} (after pre-concentration)	

Table S2. Composition of simulated Hanford LAW melter recycle stream.

Anion	Concentration(mol/L)	Anion: TcO_4^- molar ratio
TcO_4^-	1.94×10^{-4}	1.0
NO_3^-	6.07×10^{-2}	314
Cl^-	6.39×10^{-2}	330
NO_2^-	1.69×10^{-1}	873
SO_4^{2-}	6.64×10^{-6}	0.0343
CO_3^{2-}	4.30×10^{-5}	0.222

Table S3. Summary of X-Ray Crystallographic Data.

Complexes	[**Ru3**]$(ReO_4)_2$
**Empirical formula**	$C_{204}H_{138}N_{24}O_{24}Re_6Ru_3$
**Formula weight**	4729.79
**Temperature/K**	193.00
**Crystal system**	monoclinic
**Space group**	$P2_1/c$
**Unit cell dimensions**	a = 31.456(17) Å, α = 90°<br>b = 24.499(13) Å, β = 106.554(9)°<br>c = 29.263(16) Å, γ = 90°
**Volume/Å^3**	21616(20)
**Z**	4
**ρ_{calc}g/cm$_3$**	1.453
**μ/mm^{-1}**	3.612
**F(000)**	9216.0
**Crystal size/mm^3**	0.12 × 0.1 × 0.09
**Radiation**	MoKα (λ = 0.71073)
**2Θ range for data collection/°**	3.906 to 49.424
**Index ranges**	-37≤ h ≤ 37, -28 ≤ k ≤ 28, -34≤ l ≤ 34
**Goodness-of-fit on F^2**	1.230
**Final R indexes [I>=2σ (I)]**	R_1 = 0.1494, wR_2 = 0.3459
**Final R indexes [all data]**	R_1 = 0.1774, wR_2 = 0.3604
**Largest diff. peak/hole /eÅ^{-3}**	1.76/-1.71

3. References

1. A. Hazra, C. Ghosh, F. Banerjee and S. K. Samanta, Highly efficient main-chain cationic polyelectrolytes for selective sensing of permanganate, perrhenate, and heparin. *ACS Appl. Polym. Mater.*, 2024, **6**, 6540-6551.
2. C.-P. Li, H. Zhou, J. Chen, J.-J. Wang, M. Du and W. Zhou, A highly efficient coordination polymer for selective trapping and sensing of perrhenate/pertechnetate. *ACS Appl. Mater. Interfaces*, 2020, **12**, 15246-15254.
3. S. Rapti, S. A. Diamantis, A. Dafnomili, A. Pournara, E. Skliri, G. S. Armatas, A. C. Tsipis, I. Spanopoulos, C. D. Malliakas and M. G. Kanatzidis, Exceptional TcO_4^- sorption capacity and highly efficient ReO_4^- luminescence sensing by Zr^{4+} MOFs. *J. Mater. Chem. A*, 2018, **6**, 20813-20821.
4. Q.-H. Hu, X. Gao, Y.-Z. Shi, R.-P. Liang, L. Zhang, S. Lin and J.-D. Qiu, Tailor-made multiple interpenetrated metal-organic framework for selective detection and adsorption of ReO_4^-. *Anal. Chem.*, 2022, **94**, 16864-16870.
5. G. Singh, S. P. Pandey and P. K. Singh, A dual intensity and lifetime based fluorescence sensor for perrhenate anion. *Sens. Actuators, B*, 2021, **330**, 129346.
6. S. Khan and S. K. Mandal, Luminescent 2D pillared-bilayer metal-organic coordination networks for selective sensing of ReO_4^- in water. *ACS Appl. Mater. Interfaces*, 2021, **13**, 45465-45474.
7. X.-R. Chen, C.-R. Zhang, X. Liu, R.-P. Liang and J.-D. Qiu, Ionic covalent organic framework for selective detection and adsorption of TcO_4^-/ReO_4^-. *Chem. Commun.*, 2023, **59**, 9521-9524.
8. J.-X. Qi, C.-R. Zhang, X.-J. Chen, S.-M. Yi, C.-P. Niu, J.-L. Liu, L. Zhang, R.-P. Liang and J.-D. Qiu, 3D ionic olefin-linked conjugated microporous polymers for selective detection and removal of TcO_4^-/ReO_4^- from wastewater. *Anal. Chem.*, 2022, **94**, 10850-10856.
9. S.-M. Yi, C.-R. Zhang, W. Jiang, X. Liu, C.-P. Niu, J.-X. Qi, X.-J. Chen, R.-P. Liang and J.-D. Qiu, Ionic liquid modified covalent organic frameworks for efficient detection and adsorption of ReO_4^-/TcO_4^-. *J. Environ. Chem. Eng.*, 2022, **10**, 107666.
10. M. R. Choi and B. Lee, Synthesis of cationic carbon quantum dot-based dual emission fluorescence sensor for detecting perrhenate anions in aqueous solutions. *Opt. Mater.*, 2022, **134**, 113190.
11. V. Amendola, G. Bergamaschi, M. Boiocchi, R. Alberto and H. Braband, Fluorescent sensing of ^{99}Tc pertechnetate in water. *Chem. Sci.*, 2014, **5**, 1820-1826.
12. B. P. Aryal, P. Brugarolas and C. He, Binding of ReO_4^- with an engineered MoO_4^{2-}-binding protein: towards a new approach in radiopharmaceutical applications. *JBIC, J. Biol. Inorg. Chem.*, 2012, **17**, 97-106.
13. H.-L. Xu, J.-Y. Zhang, J.-S. Su, L. Li, H.-Y. Cao, G.-L. Li and Z.-H. Ni, Efficient capture and dual-model fluorescence detection of ReO_4^- using a TPE-based pyridinium network. *Dyes Pigm.*, 2025, **235**, 112579.
14. J. Y. Lim and P. D. Beer, Superior perrhenate anion recognition in water by a halogen bonding acyclic receptor. *Chem. Commun.*, 2015, **51**, 3686-3688.
15. S. P. Pandey, A. M. Desai and P. K. Singh, A highly sensitive fluorescence “turn on” detection of perrhenate Anion, a non-radioactive surrogate of hazardous pertechnetate anion. *Sens. Actuators, B*, 2020, **323**, 128675.

16. A. M. Desai and P. K. Singh, An ultrafast molecular-rotor-based fluorescent turn-on sensor for the perrhenate anion in aqueous solution. *Chem. - Eur. J.*, 2019, **25**, 2035-2042.
17. A. M. Desai and P. K. Singh, Ratiometric fluorescence turn-on sensing of perrhenate anion, a non-radioactive surrogate of hazardous pertechnetate, in aqueous solution. *Sens. Actuators, B*, 2018, **277**, 205-209.
18. S. Chatterjee, A. E. Norton, M. K. Edwards, J. M. Peterson, S. D. Taylor, S. A. Bryan, A. Andersen, N. Govind, T. E. Albrecht-Schmitt and W. B. Connick, Highly selective colorimetric and luminescence response of a square-planar platinum(II) terpyridyl complex to aqueous TcO_4^-. *Inorg. Chem.*, 2015, **54**, 9914-9923.
19. D.-Y. Xu, L. Chen, X. Dai, B.-Y. Li, Y.-X. Wang, W. Liu, J. Li, Y. Tao, Y.-L. Wang, Y. Liu, G.-W. Peng, R.-H. Zhou, Z.-F. Chai, S.-A. Wang, A porous aromatic framework functionalized with luminescent iridium(III) organometallic complexes for turn-on sensing of $^{99}TcO_4^-$. *ACS Appl. Mater. Interfaces*, 2020, **12**, 15288-15297.
20. S. Mondal, A. Rashid and P. Ghosh, A pentafluorophenyl functionalized RuII-probe having halogen bond center toward recognition and sensing of perrhenate and dihydrogen phosphate. *J. Organomet. Chem.*, 2021, **952**, 122027.
